# Rethinking future flood hazard: Hourly data challenge daily flood projections in Alpine catchments

**DOI:** 10.1126/sciadv.aed6012

**Published:** 2026-07-15

**Authors:** Paul C. Astagneau, Raul R. Wood, Manuela I. Brunner

**Affiliations:** ^1^ WSL Institute for Snow and Avalanche Research SLF, Davos Dorf, Switzerland.; ^2^ Institute for Atmospheric and Climate Science, ETH Zürich, Zürich, Switzerland.; ^3^ Climate Change, Extremes and Natural Hazards in Alpine Regions Research Center CERC, Davos Dorf, Switzerland.

## Abstract

This study challenges a common misconception in current flood hazard research that floods will decrease under a warming climate in many rivers due to changes in land-surface processes, particularly decreases in snowmelt. This view persists because most studies on flood changes rely on daily resolution data, which masks flood changes due to subdaily rainfall intensification. We show that daily streamflow projections systematically underestimate flood changes compared with hourly projections in Alpine catchments. Our results highlight that the sign of change can even switch from negative to positive in strongly snow-influenced catchments when taking an hourly perspective because intensified subdaily precipitation can outweigh snowmelt decline. These results highlight that using daily instead of hourly projections may lead to wrong conclusions on both the magnitude and direction of flood changes. An hourly resolution perspective is, therefore, crucial to reliably guide adaptation strategies to extreme Alpine floods in a warming world.

## Teaser

Hourly data challenge the common assumption that floods will decrease in a warming climate in the Alps.

## INTRODUCTION

In the Alps, floods evolve over different timescales from a few hours to several days and have diverse generation mechanisms (*1*, *2*), including convective storms in summer, prolonged rainfall on saturated soils, and a combination of rainfall and snowmelt (*3*–*5*). As rainfall intensities are increasing with climate change (*6*, *7*), especially at subdaily timescales (*8*–*10*), we expect Alpine floods to intensify. However, land-surface processes that control river flows such as snow accumulation, melt, and soil moisture before floods are mostly decreasing (*11*–*13*). As a result, rainfall intensification does not necessarily translate to higher flood peaks (*14*–*16*), particularly in snow-dominated regions where decreases in snowmelt contributions can partly compensate for increases in rainfall intensities (*17*–*20*). Overall, the combined effects of changes in rainfall extremes, snow, and soil moisture on flood hazard in a warming climate remain poorly understood and quantified.

Although streamflow can react quickly to subdaily rainfall extremes by developing into flood peaks, most climate impact assessments focusing on floods rely on daily resolution instead of hourly data (*21*–*23*). This is because of the availability and quality of daily compared with hourly data (*24*). Most of these assessments, based on daily resolution, project that flood peaks likely either do not change (*21*, *25*, *26*) or even decrease (*19*, *27*, *28*) in many snow-influenced catchments because of the decreasing contribution of snowmelt to streamflow (*18*–*20*, *29*). This effect induces a shift toward earlier high flows (*21*, *25*, *27*). Such daily resolution assessments could potentially provide a distorted picture of future flood changes because hourly rainfall extremes are increasing faster than daily extremes in a warming climate (daily extremes increase at ≤7%/°C warming rates compared with 7 to 14%/°C for subdaily extremes) (*6*, *7*, *30*, *31*). These stronger increases in hourly rainfall extremes might in future outweigh the decreases in snowmelt contributions. The few studies using hourly instead of daily resolution data for flood projections suggest that increases in rainfall in some snow-influenced catchments may result in increases rather than decreases in flood peaks (*32*–*35*). Furthermore, potential flood change signals can be undistinguishable from noise in many cases because rainfall extremes are subject to large interannual variations due to internal climate variability (*35*–*37*). Therefore, the apparent contradiction between the increase in subdaily rainfall extremes and the decrease in flood magnitudes needs to be investigated in the light of time resolution and internal climate variability.

Using a dataset of 384 catchments in the Alps, namely, Switzerland and Austria, we investigate the sensitivity of flood change signals to the choice of time resolution. We show that the widely used daily perspective could lead to wrong conclusions on future flood changes, especially in snow-dominated areas. We show that, under the RCP8.5 (Representative Concentration Pathway 8.5) scenario (*38*) for precipitation and temperature changes, and, in the light of internal climate variability, the declining flood magnitudes in snow-influenced catchments projected at daily resolution do not hold at hourly resolution. Instead, the hourly perspective reveals a consistent increase in extreme flood peaks in most mountain catchments for the RCP8.5 scenario. This key finding highlights that increases in subdaily rainfall extremes can compensate for declines in snowmelt contributions in many snow-influenced catchments. It also highlights that taking a daily perspective in climate impact assessments on floods is misleading and that an hourly perspective is crucial to reliably inform adaptation strategies to extreme floods in a warming world in the Alps.

## RESULTS

### Diverging trends in flood magnitude and frequency depending on time resolution

**Fig. 1.**
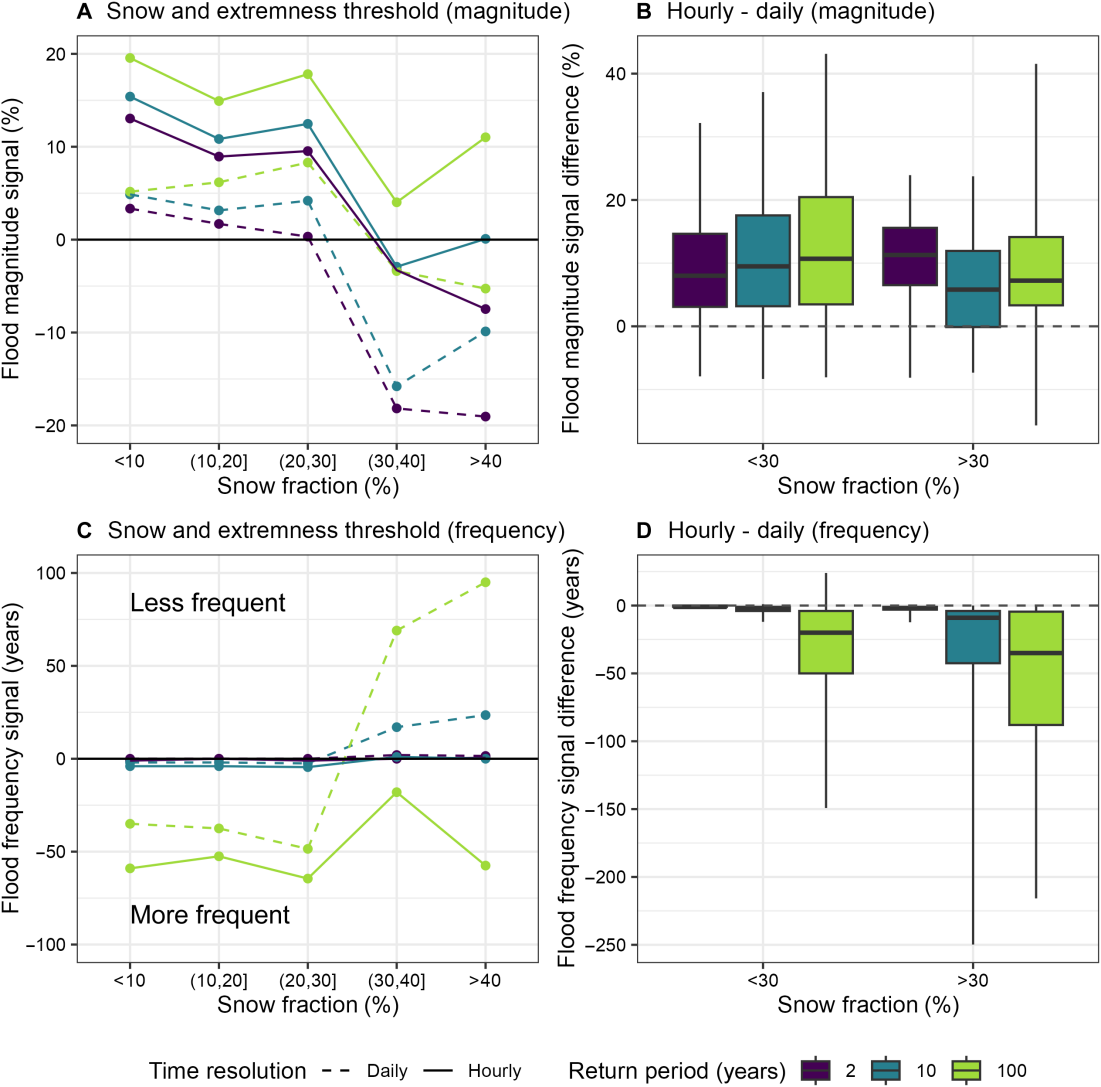
Comparison between hourly and daily flood magnitude and frequency change signals for 384 catchments in the Alps (2070–2099 compared with 1991–2020). (**A** and **C**) The median flood magnitude and frequency change signals across catchments grouped by snow fraction. (**B** and **D**) The catchment differences in the magnitude and frequency change signals between hourly and daily projections for weakly (snow fraction of <30%) and strongly snow-influenced catchments (snow fraction of >30%). The sample size for the different snow fraction classes is reported in fig. S1B.

Our comparison of daily with hourly flood projections for the RCP8.5 scenario (2070–2099 compared with 1991–2020) for 384 catchments in the European Alps (Switzerland and Austria) shows that daily resolution projections underestimate change signals in flood magnitude compared with hourly projections for more than 75% of the catchments (Fig. 1, A and B).

The projected changes and differences between hourly and daily resolution flood estimates for three different return periods (2, 10, and 100 years) differ between weakly (snow fraction of <30%) and strongly snow-influenced catchments (snow fraction of >30%; Fig. 1A). Daily simulations project a decrease in flood magnitudes in strongly snow-influenced catchments (median trends from −19 to −4%, significant in more than 50% of catchments; fig. S5), whereas hourly projections show either a weaker decrease or even an increase in flood magnitudes (median trends from −7 to +11%, significant in 30 to 60% of catchments; fig. S4). In weakly snow-influenced catchments, daily simulations project a median increase in flood magnitudes from +4 to +8% (significant in 45 to 75% of catchments; fig. S4), whereas hourly simulations project a median increase from +9 to +19% (significant in 70 to 95% of catchments; fig. S4). The median difference in flood magnitude trends derived from daily versus hourly projections is 10 and 8% in weakly versus strongly snow-influenced catchments, respectively (Fig. 1B). While the difference in daily versus hourly flood magnitude trends depends on the degree of snow influence and therefore elevation, we found no relationship between daily versus hourly flood magnitude trends and catchment area (fig. S6).

The projected changes and the differences between hourly and daily resolution projections differ for moderate (2- and 10-year) and extreme (100-year) flood events (Fig. 1A). Daily simulations project a decrease in flood magnitudes in strongly snow-influenced catchments for moderate (median trend of −19 to −10%, significant in 75 to 95% of catchments; fig. S5) and extreme flood events (median trend of −4%, significant in 50 to 65% of catchments; fig. S5), whereas hourly projections show an increase in flood magnitudes for extreme flood events (median trend of +7%, significant in about 60% of catchments; fig. S4) and a weaker decrease for moderate flood events (median trends from −7 to 0%, significant in 50 to 75% of catchments; fig. S5). In weakly snow-influenced catchments, daily simulations project a median increase in flood magnitudes from 1 to 5% for moderate flood events (significant in 45 to 70% of catchments; fig. S4) and from 5 to 8% for extreme flood events (significant in 65 to 75% of catchments; fig. S4), whereas hourly simulations project an increase from 9 to 15% for moderate events (significant in 70 to 95% of catchments; fig. S4) and from 15 to 19% for extreme events (significant in 90 to 95% of catchments; fig. S4).

The change in flood frequency also reveals substantial discrepancies between hourly and daily projections, particularly for extreme flood events in strongly snow-influenced catchments (Fig. 1, C and D). In these catchments, daily simulations project that extreme floods (100-year flood) become less frequent (170- to 195-year flood; median) in the future, whereas they become more frequent (80- to 45-year flood) according to the hourly simulations. Daily simulations also suggest that moderate floods (10-year flood) will become less frequent in these catchments, which occur every 30 years, whereas hourly simulations show no change in frequency. In weakly snow-influenced catchments, both daily and hourly projections show an increase in the 100-year flood event frequency. However, hourly projections show a considerably larger increase in frequency (from 100- to 40-year flood) compared with daily projections (from 100- to 60-year flood). The median difference in the 100-year flood frequency change signal between daily and hourly resolution is 20 and 40 years in weakly versus strongly snow-influenced catchments, respectively. The large variability in frequency signal differences across catchments shows that many strongly snow-influenced catchments experience a marked reduction in flood occurrence when using daily simulations compared with using hourly simulations.

### A significant divergence between daily and hourly flood projections reached in the first half of the 21st century

**Fig. 2.**
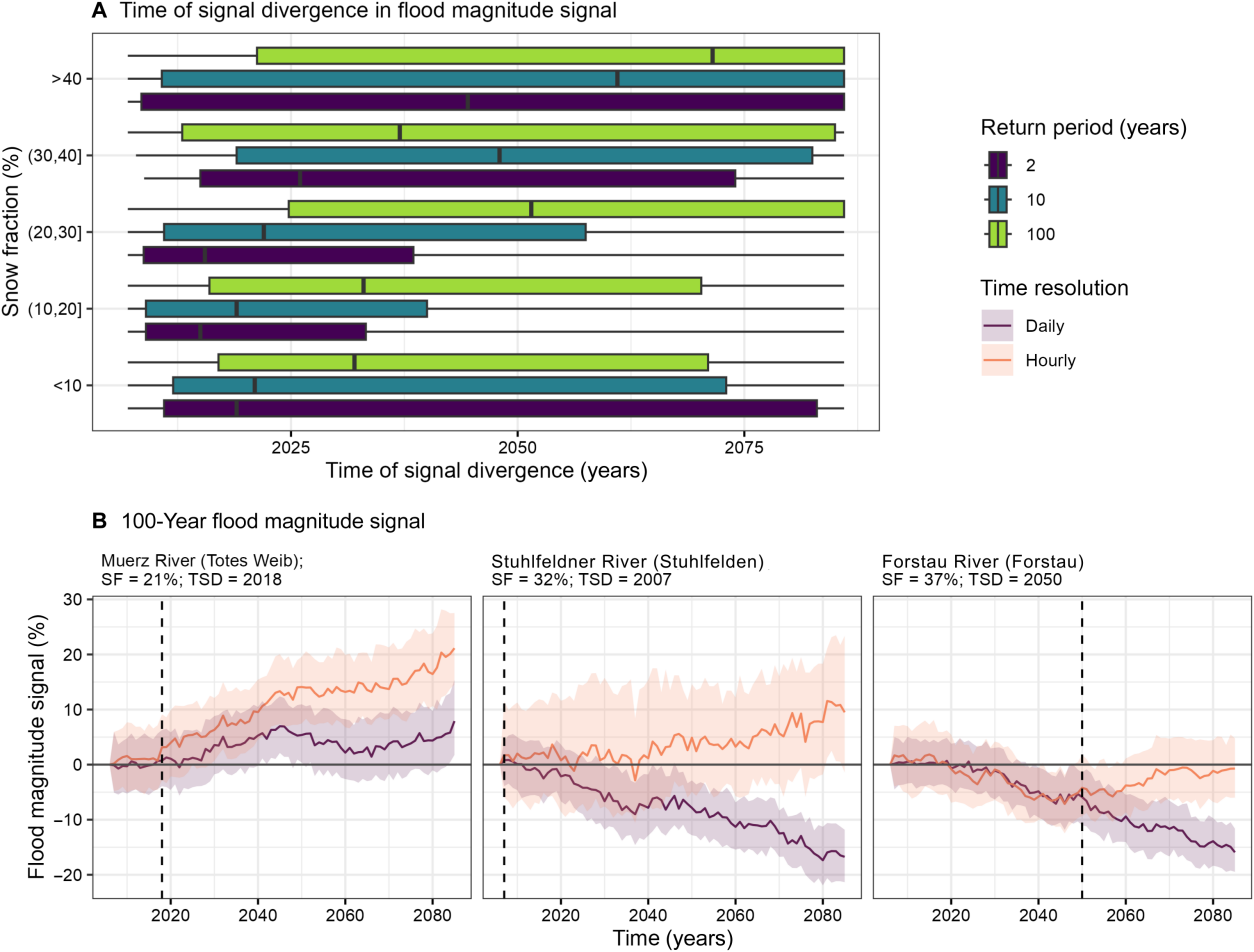
Emergence of flood change signal difference between hourly and daily projections. (**A**) Time of signal divergence (TSD) between daily and hourly flood projections across return periods and snow fractions for 384 catchments. (**B**) Three examples of transient 100-year daily and hourly flood magnitude projections: Muerz, Stuhlfeldner, and Forstau Rivers (see fig. S1A for location). The time at which a significant difference emerges between the daily and hourly projections is indicated by a dark dashed vertical line. The solid lines represent the median magnitude change signals for daily and hourly projections. The confidence interval around the median is the 66% interval. The sample size for the different snow fraction (SF) classes is reported in fig. S1B.

We now investigate when the daily and hourly flood signals significantly diverge from each other for the RCP8.5 scenario. By explicitly accounting for internal climate variability, we can robustly identify the year when daily and hourly flood magnitude change signals start to diverge (time of signal divergence; Fig. 2). The differences between hourly and daily flood projections are significant for most catchments and all return periods (the time of signal divergence is before the end of the century for more than 75% of catchments; Fig. 2A). In weakly snow-influenced catchments, these differences emerge earlier (before mid-century for the 100-year flood) in catchments with little to no snow (snow fraction of <20%) than in those with more snow (after mid-century for the 100-year flood for catchments with snow fractions between 20 and 30%; significant). We also observe an earlier time of signal divergence for moderate flood events than for extreme floods for these catchments (snow fraction of <30%; significant). In strongly snow-influenced catchments, the differences between hourly and daily projections emerge later than in weakly snow-influenced catchments for moderate (significant for 2- and 10-year events) but not extreme flood events (i.e., not significant). In strongly snow-influenced catchments, the time-of-signal-divergence distributions span a large range of years across catchments. For example, for the 100-year flood and catchments with snow fractions between 30 and 40%, the first quartile of time of signal divergence is 2012 and the third quartile is 2083. This indicates substantial differences in time of signal divergence between catchments within one snow fraction group.

We illustrate these differences using three examples of a transient change signal in the 100-year flood magnitude (Fig. 2B). For the Muerz River, which is a weakly snow-influenced catchment (21% snow fraction), the daily and hourly signals diverge significantly from 2018 onward. Daily and hourly projections indicate an increase in the 100-year flood magnitude before mid-century, reaching median values of +8% (daily) and +21% (hourly), respectively. The daily and hourly flood signals of the Stuhlfeldner River, where snow strongly influences streamflow (32% snow fraction), diverge significantly already in 2007. The daily signal shows a continuous decrease in flood magnitude, reaching a median value of −16% by the end of the century, whereas the hourly signal shows no change until 2040, followed by an increase to +10% by the end of the century. The third example, the Forstau River, which is similarly strongly snow influenced (37% snow fraction), shows a decrease in flood magnitude from a daily perspective (−15% by the end of the century), whereas the hourly perspective shows a decrease followed by an increase reaching no changes by the end of the century. The daily and hourly signals start diverging significantly after mid-century (2050).

### Weaker shift in flood seasonality projected by hourly compared with daily simulations in strongly snow-influenced catchments

**Fig. 3.**
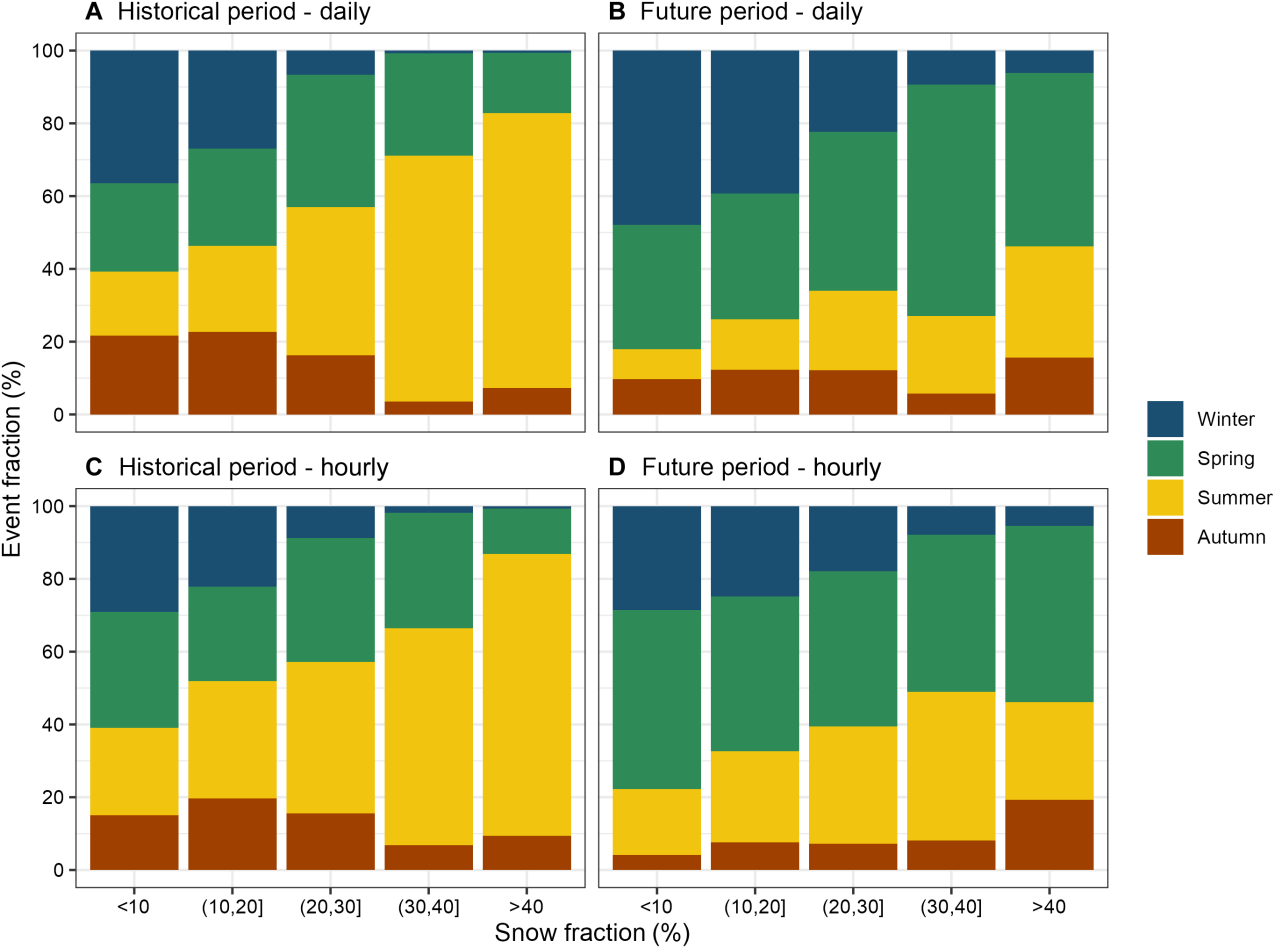
Changes in the fraction of events per season. Fraction of events for the historical period (1991–2020) (**A** and **C**) and the future period (2070–2099) (**B** and **D**) across time resolutions (daily versus hourly) and groups of catchments with different snow fractions.

**Fig. 4.**
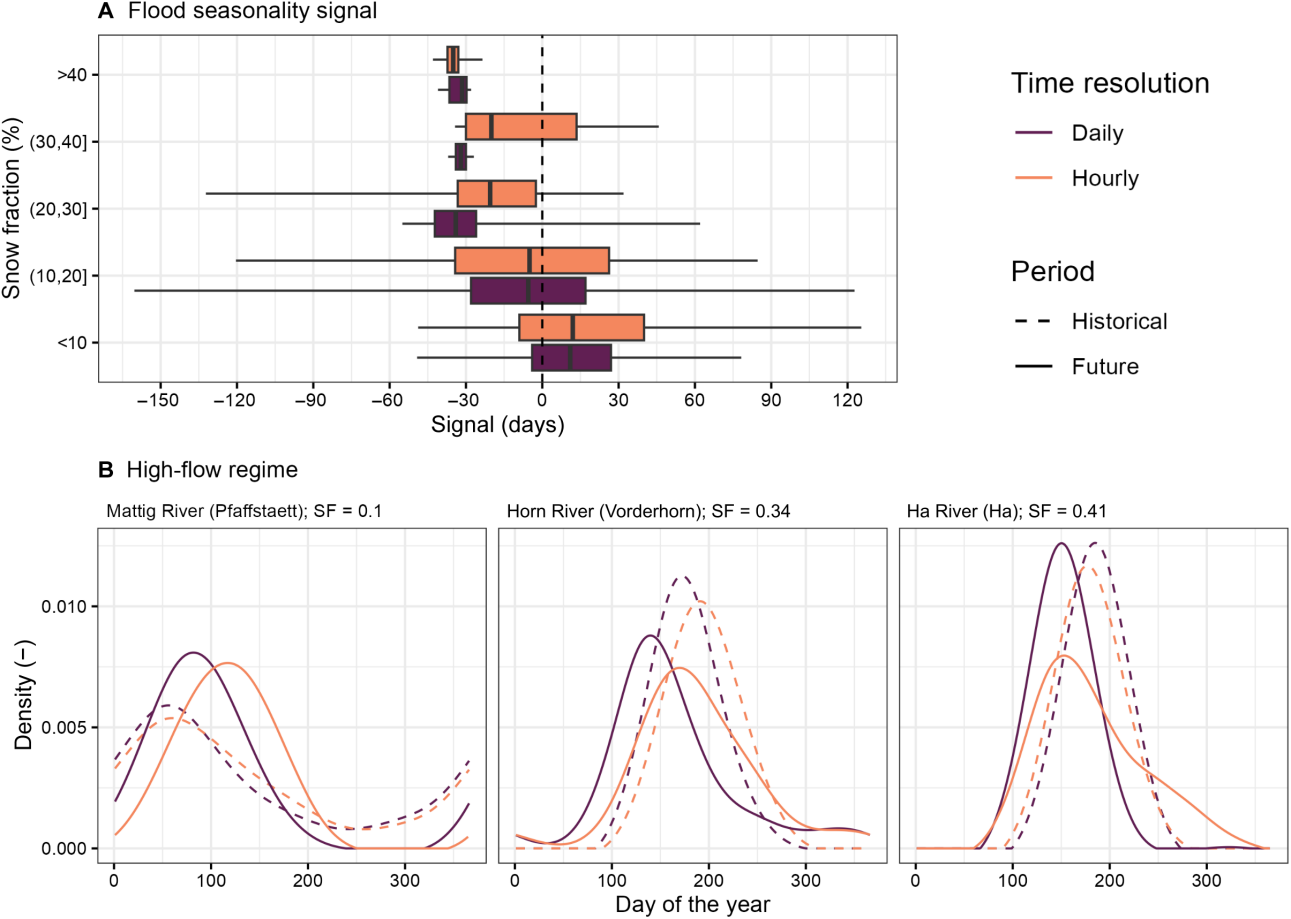
Changes in flood seasonality (2070–2099 compared with 1991–2020). (**A**) Change signal in the main flood season across time resolutions (daily versus hourly) and groups of catchments with different snow fractions. (**B**) Three examples of a mismatch between flood seasonality trends derived from daily and hourly resolution projections: Mattig, Horn, and Ha Rivers (see fig. S1A for location). The sample size for the different snow fraction (SF) classes is reported in fig. S1B.

Next, we examine how these divergences in flood magnitude projections translate to variations in flood seasonality between hourly and daily projections until the end of the century (Figs. 3 and 4). We find that the dominant flood season shifts from summer to spring in strongly snow-influenced catchments, independently of the time resolution considered (Fig. 3). This shift toward floods earlier in the year in the catchments with >20% snow fraction differs between the hourly and daily projections: It is weaker when taking the hourly compared with the daily perspective (Fig. 4A; significant). While daily simulations project a shift of 35 days (median) in snow-influenced catchments, hourly simulations project a shift of 20 days in catchments with a snow fraction between 20 and 40%. Very strongly snow-influenced catchments (snow fractions of >40%) exhibit similar seasonal shifts from summer to spring for both time resolutions. Hourly projections also indicate substantial differences in flood seasonal shift across snow-influenced catchments compared with daily projections, which instead show a consistent shift in the flood season of more than a month earlier for most of the catchments. This divergence between daily and hourly resolution is further emphasized by the fraction of events that occur in each season (Fig. 3). While flood seasonality is similar for both time resolutions in the historical period, it differs in the future period in snow-influenced catchments. For catchments with a snow fraction between 30 and 40%, the fraction of events occurring in summer and autumn is 49% in the hourly projections compared with 27% in the daily projections. Overall, the hourly projections suggest that flood seasonality in snow-influenced catchments might shift from a snowmelt-dominated regime in summer to a bi-modal snowmelt- and rainfall-dominated regime in spring and summer, respectively. For catchments with lower snow fractions (10 to 20%), daily and hourly projections show no clear seasonal shifts, with large variability across the catchment set. However, in catchments with little snow influence (<10%), daily projections suggest a transition from a nonseasonal flood regime to a winter-dominated one, whereas hourly projections suggest a transition to a spring-dominated flood regime.

We illustrate some of the catchment-specific differences using three examples (Fig. 4B). For the Mattig River, a catchment with limited snow influence (snow fraction of 10%), the flood season shifts from early to late winter for both resolutions, with a stronger shift for the hourly resolution. In contrast, the two snow-influenced catchments show a very different picture. In the historical period, their main flood season occurs in spring and early summer, when snow melts and most rainfall storms occur. Both time resolutions indicate a similar flood season pattern for this period, although the hourly resolution shows a slightly later flood season for the Horn River (snow fraction of 34%). However, the daily resolution indicates an earlier flood season in the warmer period for both catchments, whereas the hourly resolution shows a different trend pattern. Hourly projections suggest that the dominant flood season may not change substantially and that floods could occur at different times of the year. For the Ha River (snow fraction of 41%), hourly simulations project a similar shift in the dominant flood season to that shown by daily projections. However, the daily projections, in contrast to the hourly ones, do not show that a large number of floods could also occur in late summer and early autumn compared with the historical period.

### Increases in extreme precipitation outweigh snowmelt decreases at hourly but not daily resolution

**Fig. 5.**
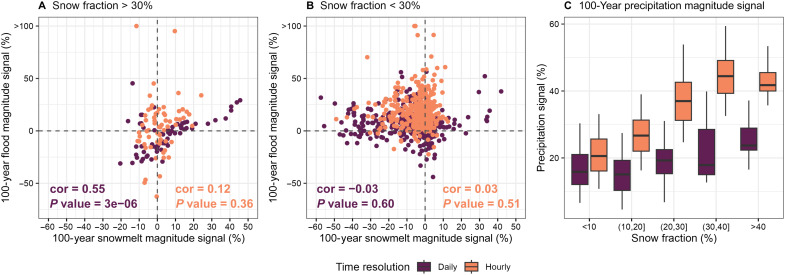
Drivers of differences between daily and hourly flood projections. (**A**) Comparison between 100-year flood magnitude and 100-year snowmelt magnitude change signal for daily and hourly projections for strongly snow-influenced catchments (snow fraction of >30%; *n* = 64) and (**B**) weakly snow-influenced catchments (snow fraction of <30%; *n* = 320). (**C**) Comparison between daily and hourly 100-year precipitation signals across classes of catchments with different snow fractions. The change signals are shown for the period 2070–2099 compared with the period 1991–2020. cor is the Pearson correlation coefficient.

Last, we examine potential reasons for the discrepancies between hourly and daily flood projections. Specifically, we investigate the relationship between the change signal in the magnitude of snowmelt extremes compared with that of flood extremes (Fig. 5, A and B) and the magnitude signal of precipitation extremes across time resolutions and snow fraction catchment groups (Fig. 5C). We show that the change signal of snowmelt extremes drives the change signal of extreme floods at daily resolution (strong correlation) in strongly snow-influenced catchments (Fig. 5A), but not at the hourly resolution (the orange points are scattered and the correlation is weak) and not in weakly snow-influenced catchments (Fig. 5B). At daily resolution, in strongly snow-influenced catchments, the 100-year flood magnitude signal is almost always below zero, except when the 100-year snowmelt magnitude signal is large. However, note that seven catchments show an increase in the 100-year flood magnitude signal but a decrease in the 100-year snowmelt signal. Except for these catchments, the stronger the snowmelt change signal, the stronger the flood change signal. This pattern is not present in the hourly signal, suggesting that, on hourly timescales, different processes are changing and drive the flood signal than at daily resolution, even in strongly snow-influenced catchments.

This disconnection between changes in floods and snowmelt extremes at hourly resolution may be due to hourly precipitation intensification (Fig. 5C). We find that hourly precipitation extremes intensify more strongly than daily precipitation extremes, especially in snow-influenced catchments (median increase of >40% for hourly and >20% for daily). These results confirm findings of previous studies (*6*, *8*) and suggest that, contrary to what the daily perspective might imply, snowmelt extremes are not the only drivers of flood changes in snow-influenced catchments. Instead, the intensification of subdaily precipitation extremes also plays a substantial role in generating extreme floods, which are missed when looking at daily scales.

## DISCUSSION

Our results show that the flood change signal is highly sensitive to the choice of time resolution in the Alps. For the RCP8.5 scenario, the hourly flood projections show that extreme floods are likely to increase in a warming climate because of intensified subdaily rainfall, rather than decrease due to changes in land-surface processes such as declining snowmelt in strongly snow-influenced catchments (Fig. 1). These results disagree with existing literature on the subject (*19*, *28*, *39*), which consistently report a decline or no change in floods in snow-influenced, including Alpine, catchments. We demonstrated that this discrepancy is likely due to the daily (i.e., coarse) time resolution commonly used in these studies, which distorts our perception of flood changes. The daily resolution overemphasizes the buffering effect of declining snowmelt (*17*, *19*, *20*) while masking the flood response to intensifying subdaily rainfall (Fig. 5).

While it is well established that daily data underestimate instantaneous flood peaks (*40*), assuming a fixed ratio between daily and hourly peaks during the historical period, as is sometimes done when only daily data are available (*40*–*42*), would not resolve the discrepancy between hourly and daily flood projections because the relationship between daily and subdaily peaks is nonstationary, as demonstrated by our analyses. These findings have important implications for flood design studies that rely on such approaches to estimate instantaneous peaks (*40*).

The differences between hourly and daily projections are significant for most catchments and the change signals diverge from each other before mid-century in more than 50% of the catchments (Fig. 2). This result corroborates results of recent studies, indicating that increasing hourly heavy rainfall has already resulted in increasing flood peaks in the Alps over the past 60 years (*7*) and in small catchments compared with larger catchments in Europe (*43*). However, we did not find any relationship between flood magnitude trends and catchment area in our study. Our results also are in line with a recent study that projected an increase in the flashiness of floods in the conterminous United States in a warmer climate (*44*). However, we also demonstrated substantial variability in flood changes and differences between hourly and daily resolutions across the dataset (Figs. 1 and 2). This variability highlights that substantial uncertainties remain regarding how floods will change in snow-influenced catchments in a warming climate.

Moderate flood extremes may still decrease in snow-influenced catchments in the Alps due to reduced snowmelt, albeit, to a lesser extent, than anticipated with daily projections. Most studies using flood projections have focused on moderate flood extremes because climate model ensembles do not account for internal climate variability (*19*, *21*). This could also explain why the hydrology community’s understanding of flood changes in snow-influenced catchments is limited to date, as our results show large differences in changes between moderate and extreme flood events in such catchments in the Alps. This confirms findings of previous studies that highlighted the impact of the extremeness level on the flood change signal (*34*, *43*, *45*).

In snow-influenced catchments, floods may occur earlier in the year under a warming climate due to earlier and weaker snowmelt, as reported in previous studies (*25*, *27*, *28*, *46*), but we show that, at the daily resolution, this seasonal change is exaggerated compared with hourly simulations (Figs. 3 and 4). For many snow-influenced catchments, we demonstrated that this shift in seasonality could be less pronounced, not occur at all, or could even result in a later flood season. Overall, our results indicate that floods might occur in more seasons throughout the year in the future in the Alps, suggesting a substantial shift in flood generation processes changing from events that are strongly snowmelt influenced to events that result from a combination of different processes (*22*, *35*, *47*). While very strongly snow-influenced catchments may still experience a substantial shift toward earlier floods because of earlier snowmelt, this might not imply a decrease in flood magnitudes (Figs. 1A and 4A).

Our results are based on a model chain consisting of feeding climate model output into a hydrological model. Therefore, and similar to other hydrological climate impact studies, it involves several sources of uncertainty, which we tried to constrain as well as possible by carefully selecting climate datasets and calibrating and evaluating the hydrological model. As input data, we chose a large ensemble of climate projections derived from different initial climate conditions. While this ensemble allowed us to quantify internal climate variability, it was derived from one climate model only [Canadian Regional Climate Model version 5 (CRCM5) large ensemble (CRCM5-LE); see the “Climate projections” section], meaning that we were not able to quantify climate model uncertainty. To assess how hourly and daily flood projections may differ under alternative climate models, our analysis should be extended to include other climate models that cover a wider range of potential climate outcomes once alternative high-resolution large ensembles for the European domain become available. The climate ensemble used in our study originates from a regional climate model, meaning that convection processes are parametrized rather than explicitly represented. Future studies on changes in flood peaks at the hourly timescale could, therefore, include climate model ensembles that solve convection explicitly (*48*, *49*). While these may provide further insights into flood changes generated by intensification of subdaily rainfall (*50*, *51*), they are usually only available for short time slices, which challenges the analysis of extreme flood events. Besides climate model uncertainties, hydrological modeling uncertainty can affect the reliability of our hydrological projections (*52*–*54*). To constrain hydrological modeling uncertainty, we implemented the following quality checks: (i) ensured a high level of consistency between daily and hourly calibrations (see the “Calibration” section); (ii) carefully evaluated the hydrological model simulations, including the model’s ability to simulate peaks outside the calibration period (fig. S3); (iii) assessed simulation robustness against the choice of the calibration period (fig. S8); and (iv) evaluated the performance of the hydrological model driven by climate simulations in the historical period (fig. S7). While several sources of uncertainty can affect our simulations, our analysis shows that the sensitivity of flood change signals to temporal resolution is a substantial source of uncertainty in flood projections in the Alpine region.

We demonstrated that using daily rather than hourly projections alters our understanding of how floods change in the Alps for the RCP8.5 scenario, particularly not only in snow-influenced catchments but also in low-elevation catchments. Although our study focuses on catchments in the European Alps, we believe that our findings can be generalized to other regions that experience comparable climate and hydrological changes. Therefore, future research on flood changes should adopt the hourly perspective to derive reliable flood hazard estimates.

## MATERIALS AND METHODS

We evaluated the differences between daily and hourly flood projections based on the simulations of a hydrological model driven by the temperature and precipitation projections generated by a 50-member climate model large ensemble. We based our evaluation on a flood frequency analysis to derive flood change signals, an estimation of the time of signal divergence between the daily and hourly projections, an analysis of changes in flood seasonality, and an analysis of the relationship between changes in snowmelt extremes, precipitation extremes and flood extremes. We first present the study region and the modeling frameworks and then the methods used to perform the analyses.

### Study region

We generated and studied daily and hourly flood projections for a set of 384 near-natural catchments located in the Central European Alps, specifically Switzerland and Austria (fig. S1A). These catchments were part of two publicly available large sample datasets: CAMELS-CH (*55*) and LamaH-CE (*56*). To obtain the final set of 384 catchments, we excluded catchments with large glaciers (≥10% of glacier areas) and catchments affected by large human interventions (i.e., we kept the catchments with no reservoirs for CAMELS-CH and “low influence” and “no influence” for LamaH-CE) because we do not account for these effects in our modeling framework. Additionally, we excluded catchments for which the hydrological model had a low accuracy for streamflow (31 catchments; see the “Evaluation” section). The selected catchments cover a large range of hydro-meteorological conditions present in the Alps and their surroundings. In particular, they span a large range of fractions of precipitation falling as snow (fig. S1B) and catchment areas (fig. S1C). The hydrological regimes of these catchments range from nival to pluvial, with snowmelt, antecedent wetness conditions, and rainfall extremes representing the primary flood generation processes.

### Climate projections

We used climate projections of precipitation and temperature from a single-model initial-condition large ensemble (SMILE) to account for internal climate variability, which affects the year-to-year variability of flood events and, therefore, flood frequency estimation. The SMILE projections enabled us to study long-term trends of extreme floods by allowing us to separate changes resulting from internal variability from those resulting from long-term forced trends. We used the climate projections from the 50-member CRCM5-LE from the ClimEx experiment (*57*). The CRCM5-LE was dynamically downscaled from the driving 50-member CanESM2 large ensemble (Canadian Earth System Model version 2 large ensemble) (*58*, *59*) to a spatial resolution of 0.11° (≈12 km) and a temporal resolution of 1 hour for precipitation and 3 hours for temperature. All 50 members in the driving CanESM-LE were driven with observed anthropogenic (greenhouse gases emissions, aerosols, and land cover) and natural forcings (solar and volcanic influences) over the period (1950–2005) and with the RCP8.5 scenario (*38*) from 2006 to 2099. Variations among the individual CRCM5 ensemble members stem from differences in macro- and microscale initialization within the CanESM2-LE driving model, and these can be understood as manifestations of natural climate variability. The CRCM5-LE ensemble projects an intensification of hourly precipitation extremes at a 6 to 7%/°C warming rate in the Alps [see figure 6 of (*60*)], which is aligned with the changes projected by convection-permitting models (*49*). We adjusted the biases in the precipitation and temperature projections of CRCM5-LE over Austria and Switzerland following (*61*) using the cumulative distribution function–transform (CDF-t) (*62*) univariate statistical method combined with the ensemble adjustment method (*63*), to preserve (i) the projected temperature and precipitation changes, (ii) the internal climate variability, and (iii) the dependence between precipitation and temperature simulated by the climate model (*61*). For this adjustment, we used the Swiss (RhiresD and TabsD) (*64*–*67*) and Austrian (SPARTACUS) (*68*–*70*) national daily gridded meteorological datasets over the historical period (1991–2020). For SPARTACUS, we derived the daily mean temperature from the average between minimum and maximum temperatures because mean temperature is not provided. We adjusted the biases of the climate projections at the native spatial resolution of the model (0.11°) to avoid introducing artifacts resulting from additional downscaling (e.g., overestimation of extremes and overcorrection of the drizzle effect for area means) (*71*). We only adjusted the biases at the daily time step after aggregating the 1- and 3-hour data (24-hour sum for precipitation and 24-hour average for temperature) because hourly data are not available on a long enough period to perform hourly bias adjustment (30 years) (*72*). To retrieve hourly projections, we then temporally disaggregated the daily adjusted data back to 1- and 3-hour data using the original (before bias adjustment) 1- and 3-hour anomalies to the daily mean (relative for precipitation and absolute for temperature). This procedure ensures consistency between daily and hourly projections. For temperature, we additionally linearly interpolated the resulting 3-hour temperature data to 1-hour values. Last, we calculated areal catchment precipitation and temperature for each member by area-weighted averaging of 12-km grids within the catchment outlines, resulting in 50 realizations of hourly time series of precipitation and temperature from 1991 to 2099 for each of the 384 catchments. We evaluated the performance of the bias-adjusted SMILE runs in reproducing precipitation extremes at 1- and 24-hour resolution (fig. S2). The median bias ranged from −20 to −5%. We found larger biases for hourly extremes than for daily extremes, which is consistent with adjusting the biases at the daily time step only. The biases were larger in Austrian catchments than in Swiss catchments. The biases were also larger in strongly snow-influenced catchments than mildly snow-influenced catchments in Austria, but smaller in strongly snow-influenced catchments than mildly snow-influenced catchments in Switzerland. The biases were larger in Austrian catchments than in Swiss catchments, as well as in Austria’s strongly snow-influenced catchments and Switzerland’s mildly snow-influenced catchments. Although systematic underestimation remained after bias correction, the biases were not substantial for a regional climate model at daily to hourly time steps compared with what has been reported in other studies (*73*). These results aligned with evaluations performed in (*9*) and (*74*). Furthermore, given that we calculated relative change signals, these biases should not affect our conclusions.

### Hydrological modeling

We used these bias-adjusted large-ensemble projections to generate a 50-member Hydro-SMILE ensemble by simulating streamflow at the outlet of the 384 catchments with a hydrological model. We used the hydrological model with two setups: a daily and an hourly setup. The daily setup consisted of calibrating and driving the model using only daily data (daily precipitation, temperature, and streamflow observations for calibration and daily simulations of temperature and precipitation from the climate model for hydrological projections). The hourly setup consisted of calibrating and driving the model using only hourly data, i.e., hourly observational data for calibration and bias-adjusted hourly climate projections. The use of these two setups allowed us to compare the daily projections obtained from the most common setup used in climate change impact studies with those obtained from a fully hourly setup. We used the HBV lumped rainfall-runoff model (TUW model version) (*75*, *76*) for both setups. This choice enabled us to test the hourly and daily setups based on the same model structure for a large sample of catchments and 50 ensemble members until the end of the century at reasonable computation times. The TUW model has 15 free parameters and takes time series of precipitation, temperature, and potential evapotranspiration as inputs. We estimated potential evapotranspiration using the formula by Oudin *et al.* (*77*), which uses temperature and latitude as proxies for radiation, in a similar way to the formulations by Hamon (*78*) and Hargreaves and Samani (*79*) [see also (*80*)].

#### Calibration

The 15 free parameters of the hydrological model must be calibrated to accurately simulate streamflow at the outlet of each catchment. We calibrated the model against streamflow observations using the Kling-Gupta efficiency (KGE) index (*81*) as the objective function because it emphasizes high flows and has been shown to provide superior model performance in terms of floods as compared with using alternative objective functions (*82*, *83*). We applied a warm-up period of 2 years before the calibration period to initialize the model’s internal states. Due to the differences in the time span covered by the hourly meteorological and hydrological observations in the two countries, we used different calibration periods for Switzerland and Austria. For Switzerland, we calibrated the hydrological model for the period between 2005 and 2022 and, for Austria, for the period between 2012 and 2017. To ensure consistency within a country, we used the same calibration period for the hourly and daily setups. Although the 6-year calibration period in Austria is not optimal for model calibration, leading to additional uncertainty, the primary source of uncertainty when estimating high-end return periods is likely internal climate variability (*35*, *84*). We quantify the impact of internal climate variability on the projected flood changes by using SMILE simulations, as detailed in the “Climate projections” section. Furthermore, we tested the impact of a shorter calibration period (7 years instead of 18) on the 100-yearly flood magnitude signal for the Swiss catchments and found limited differences compared with the results obtained using a longer calibration period (see fig. S8). For the daily setup, we calibrated the model on the basis of the data products used for bias adjustment (RhiresD/TabsD and SPARTACUS) and on daily streamflow observations provided through the CAMELS-CH and LamaH-CE datasets. For the hourly setup, we used hourly streamflow data provided by the Swiss Federal Office for the Environment (FOEN) (*85*) and the LamaH-CE dataset. For hourly meteorological observations, we used a combination of different products. To ensure consistency with the bias adjustment procedure, which is based on the daily precipitation data (see the “Climate projections” section), we temporally disaggregated the observed daily precipitation sums to obtain hourly precipitation. To do this, we calculated hourly relative anomalies to the daily sum using hourly precipitation data from two national datasets. We then used these hourly anomalies to disaggregate the daily sum of the daily precipitation observations. We used the CombiPrecip dataset (*86*, *87*) for Switzerland and the INCA (Integrated Nowcasting through Comprehensive Analysis) dataset (*88*, *89*) for Austria to carry out the disaggregation to subdaily timescale. CombiPrecip is based on radar measurements adjusted using precipitation gauge measurements, whereas INCA is derived from a combination of data from numerical weather forecasts, radar, and precipitation gauge measurements. For temperature data in Austria, we used the hourly data available through the INCA dataset. Hourly gridded temperature data do not exist for Switzerland. Therefore, to estimate hourly temperatures for catchments in Switzerland, we applied a spline interpolation that disaggregated daily averages to hourly averages using minimum and maximum daily temperatures.

#### Evaluation

We evaluated the streamflow accuracy of the daily and hourly model setups for the set of 384 catchments for each resolution independently. To that end, we carried out a calibration/evaluation experiment, independent from the calibration procedure described in the previous section. To perform this experiment, we calibrated the daily and the hourly models independently for two periods: 2005–2011 (P1) and 2012–2017 (P2). We evaluated the model accuracy of the first calibration run (P1) for P1 (calibration) and P2 (evaluation) and the model accuracy of the second calibration run (P2) for P2 (calibration) and P1 (evaluation). We show the results of this experiment for both the daily and hourly setups for Switzerland and for the daily setup for Austria. Due to the available time span of overlapping hourly streamflow and meteorological observations for Austria (2012–2017), we report the accuracy of the hourly calibration only between 2012 and 2017 for Austria. We evaluated model accuracy using the KGE index and the relative annual peak flow bias (APFB) (*82*). Please note that, before this analysis, we removed 31 catchments from the initial selection of 415 catchments (see the “Study region” section) because the hydrological model exhibited low streamflow accuracy (KGE of <0.5 in calibration).

The model shows satisfactory streamflow accuracy across the catchment set for both the KGE index and the APFB (fig. S3). KGE values for the daily setup in strongly snow-influenced catchments (snow fraction of >30%) during calibration range from a first quartile of 0.82 to a third quartile of 0.87 for Austria and from 0.85 to 0.89 for Switzerland. In weakly snow-influenced catchments (snow fraction of <30%), KGE values during calibration range from a first quartile of 0.75 to a third quartile of 0.82 for Austria and from 0.79 to 0.85 for Switzerland. The hourly setup shows similar values for calibration as the daily setup. For evaluation in strongly snow-influenced catchments, the KGE values of the daily setup range from a first quartile of 0.80 to a third quartile of 0.85 for Austria and from 0.78 to 0.87 for Switzerland. In weakly snow-influenced catchments, KGE values for the daily setup during evaluation range from 0.66 to 0.8 for Austria and from 0.75 to 0.84 for Switzerland. For the hourly setup, KGE values during evaluation range from a first quartile of 0.66 to a third quartile of 0.85 in strongly snow-influenced catchments and from 0.68 to 0.83 in weakly snow-influenced catchments. Overall, the KGE values are higher for strongly snow-influenced catchments than for weakly snow-influenced catchments and for Switzerland compared with Austria.

The median values of the APFB range from −25 to −20%, both in calibration and evaluation and in strongly and weakly snow-influenced catchments. For Switzerland, the model shows slightly stronger underestimation of peaks for the hourly setup compared with the daily setup (median difference of 2%) and for strongly snow-influenced catchments compared with weakly snow-influenced catchments (median difference of 2%). The accuracy of the hourly model to simulate daily peaks differed from the accuracy of the daily and hourly models to simulate daily and hourly peaks, respectively. The median daily peak flow bias of the hourly model ranges from −8 to +4%. The annual peak flow accuracy of the model across the 384 catchments is in line with results reported for other hydrological models used in large sample studies (*82*).

Additionally, we evaluated the accuracy with which the hourly hydrological model, when forced with climate simulations, reproduced observed hourly streamflow percentiles during the historical period (fig. S7). Depending on the location and the percentile, the median biases range from −30 to +20%. Biases are larger for Austrian than for Swiss catchments and for mildly snow-influenced catchments than for strongly snow-influenced ones. Because we calculate relative change signals in our analyses, the uncertainty in the change signals will not correspond to the biases in reproducing observations. If these biases are stationary, the biases in the change signals resulting from model uncertainty will be close to zero.

### Frequency analysis

Using the 50-member daily and hourly Hydro-SMILE projections for the 384 catchments, we estimated flood magnitude and frequency for a warmer future period (2070–2099) and a historical period (1991–2020) for different return periods. First, we identified events for each catchment by selecting streamflow peaks above the 99th percentile for each Hydro-SMILE member and 30-year period individually and applied a minimum time lag of 10 days between events to ensure event independence (*90*). This resulted in ∼2 events being selected per year, per ensemble member, and per catchment. We then combined all events to obtain a large sample of events (∼3000 events per catchment: 50 members × 30 years × 2 events per year). We estimated flood magnitudes corresponding to 2- to 300-year return periods using empirical probabilities (Weibull plotting position) (*91*). We presented the results for the 2-, 10-, and 100-year return periods to study both moderate flood events (2-yearly and 10-yearly) and extreme flood events (100-yearly). Using the estimated flood magnitudes for different return periods, we calculated a magnitude and a frequency change signal between the historical period and the warmer future period. We calculated a relative flood magnitude signal and an absolute flood frequency signal. To calculate the frequency signal, we matched each historical return period with the future return period that has the most similar event magnitude, as determined from the derived empirical probabilities. The frequency signal corresponds to the difference between these matched return periods. To compare the estimates derived from the daily and hourly time series, we used daily projections obtained from the daily setup and projections of daily maxima obtained from the hourly setup. For the last part of the analysis, to understand the differences between hourly and daily change signals, we applied the same frequency analysis method to calculate the magnitude signal of the 100-year precipitation event and the 100-year snowmelt event. For precipitation, we applied a minimum time lag of 2 days between events instead of 10 days to ensure independence without removing too many events (*92*).

### Time of signal divergence

To identify the period at which daily and hourly flood projections begin to diverge from each other significantly, we calculated a time of signal divergence based on the estimation of internal climate variability provided by the Hydro-SMILE projections. To do so, we combined the 50-member Hydro-SMILE projections differently than that in the “Frequency analysis” section to estimate the flood magnitudes simulated by the daily and hourly setups and corresponding to different return periods. We calculated the flood magnitudes and the corresponding internal climate variability corresponding to the 2-, 10-, and 100-year return periods for 80 climate periods, each consisting of 30 years, moving forward by 1 year from the 1991–2020 period to the 2070–2099 period. For each climate period, we randomly selected 10 Hydro-SMILE members to estimate the flood magnitudes corresponding to the different return periods using a sufficiently long period and then repeated this estimation 200 times to quantify the uncertainty resulting from internal climate variability. For each selection of 10 members, we fitted a generalized extreme value distribution (GEV) to estimate flood magnitude for each return period, using the method for event selection and independence described in the “Frequency analysis” section. We fitted an extreme value distribution because we combined 10 members instead of 50 members, which means that empirical probabilities would be less accurate than that in the “Frequency analysis” section. We used L-moments to estimate the parameters of the GEV (*93*). Repeating this selection and fitting process 200 times resulted in 200 estimates of the 2-, 10-, and 100-year flood magnitudes per catchment and climate period. Then, we calculated the magnitude change signal between each of the 80 climate periods and the historical period (i.e., first period of the 80 periods), resulting in 200 estimates of the transient magnitude change signal from the 1991–2020 period to the 2070–2099 period for each catchment and each return period. We performed this estimation for the daily setup and for the hourly setup and then tested whether the distribution of flood magnitude change signal values (consisting of 200 values) for the hourly setup was significantly different from the distribution of flood magnitude change signal values for the daily setup. To do this, we applied a two-sample Kolmogorov-Smirnov test (*94*) at a significance level of 0.05. We then defined the time of signal divergence as the middle year of the period for which the *P* value of the Kolmogorov-Smirnov test was lower than 0.05 and remained so until the end of the projections. At the end of this procedure, we obtained values of the time of signal divergence between the daily and hourly projections of the flood magnitude change signal for three return periods and 384 catchments. We also used the two-sample Kolmogorov-Smirnov test to test for significant differences between the distributions of time of signal divergence across snow fraction groups and return periods. In addition, we used the 200 estimates of the 2-, 10-, and 100-year flood magnitudes to test for the significance of the flood magnitude change signals reported in Fig. 1A, also using the two-sample Kolmogorov-Smirnov test at a significance level of 0.05. For each catchment, resolution, and return period, we tested for both lower and higher magnitudes in the future and report the corresponding distribution of *P* values in figs. S4 and S5.

### Flood seasonality

To define flood seasonality, we selected peaks in both the historical and the future period for each hydro-SMILE member using the threshold derived from the historical period. To identify the flood season, we counted the number of flood events during each day of the year, which we transformed into a density distribution. We then smoothed this distribution using a single cubic smoothing spline (*95*). We defined the flood season as the Julian day of the year associated with the highest probability of flood occurrence. We identified this flood season for each catchment and compared the estimated flood season for the future period with that of the historical period, resulting in a flood seasonality change signal. We then compared the flood seasonality change signal calculated for the daily setup with that for the hourly setup. To test for significant differences between the distributions of flood seasonality change signal across time resolutions and snow fraction groups, we applied a two-sample Kolmogorov-Smirnov test (*94*) at a significance level of 0.05. To complement the analyses, we also report the fraction of events that occur in winter (December-January-February), spring (March-April-May), summer (June-July-August), and autumn (September-October-November) for both time resolutions and for the historical and the future period.
