# Supplementary material for: Rethinking future flood hazard: Hourly data challenge daily flood projections in Alpine catchments
Source: Sci Adv. 2026 Jul 15;12(29):eaed6012. doi: 10.1126/sciadv.aed6012 (PMC13371908; doi:10.1126/sciadv.aed6012)
Supplement: Supplementary file 1 — Figs. S1 to S8 [file sciadv.aed6012_sm.pdf]

Science Advances
AAAS

Supplementary Materials for

Rethinking future flood hazard: Hourly data challenge daily flood projections in Alpine catchments

Paul C. Astagneau *et al.*

Corresponding author: Paul C. Astagneau, paul.astagneau@env.ethz.ch

Sci. Adv. **12**, eaed6012 (2026)
DOI: 10.1126/sciadv.aed6012

This PDF file includes:

Figs. S1 to S8

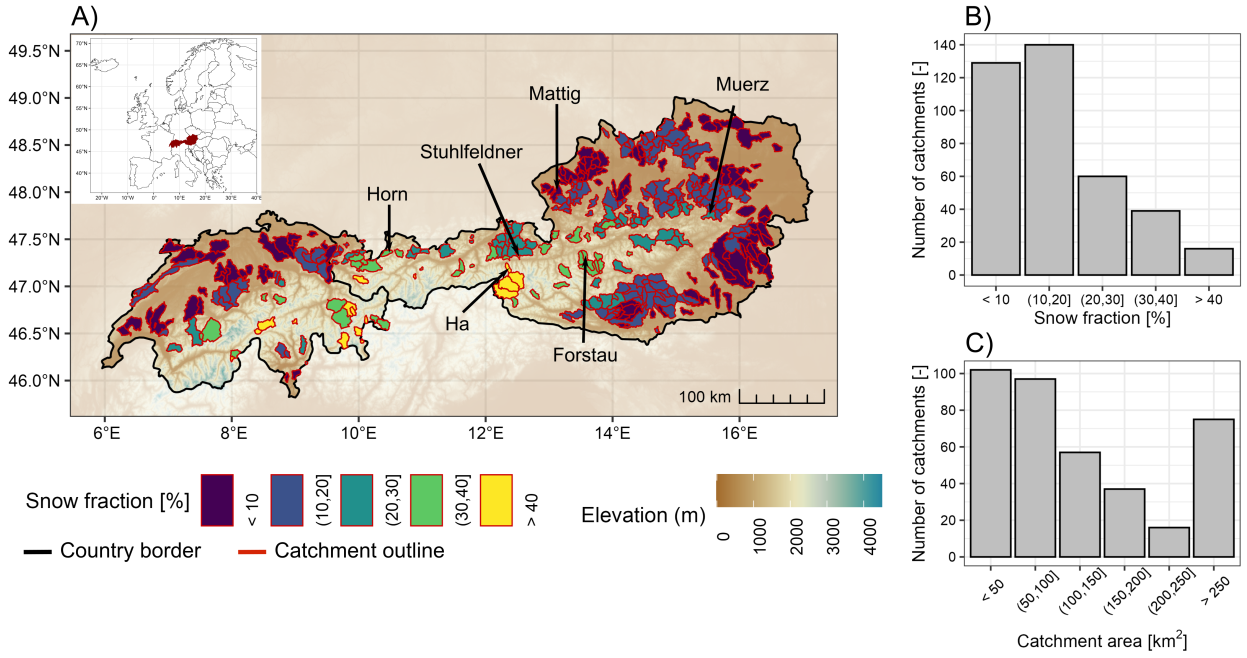

Figure S1: **Map of the catchments used for the analyses**. A) Location of the 384 catchments in Switzerland and Austria. The locations of the catchment examples shown in Figures 2B and 3B are indicated in black. B) Number of catchments for each snow fraction group. C) Number of catchments for six area groups.

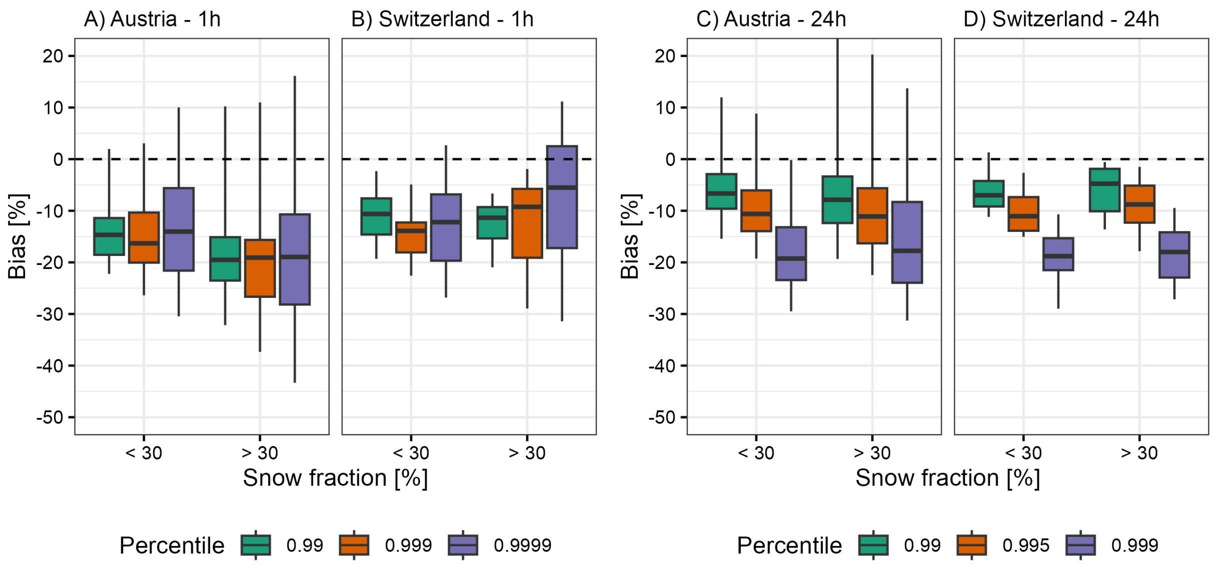

Figure S2: **Bias in precipitation extremes simulated by the climate model ensemble**. A) and B) Bias in hourly precipitation extremes. C) and D) Bias in daily precipitation extremes. The SMILE members were pooled together over the reference period to estimate the corresponding percentiles. The biases were calculated for 384 catchments.

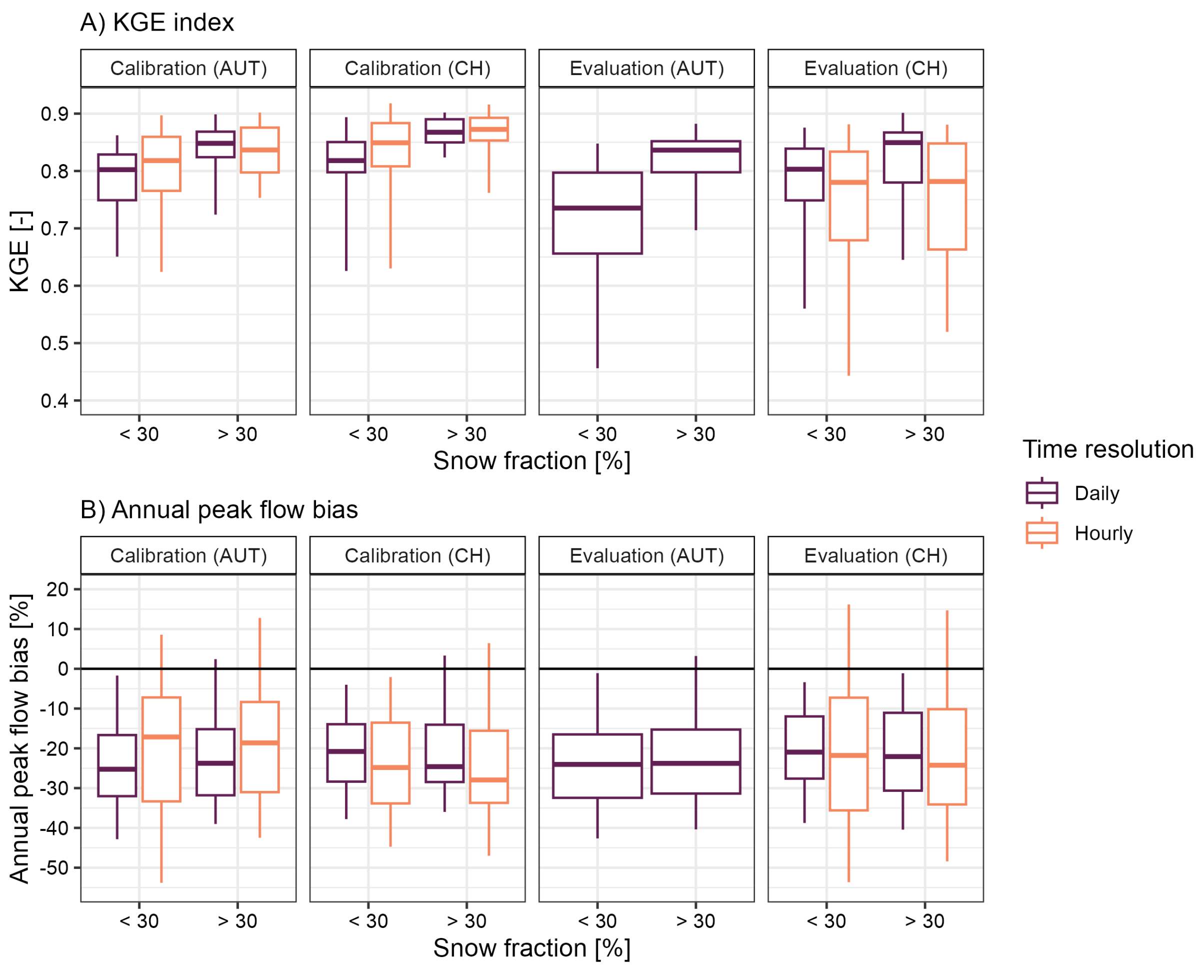

Figure S3: **Hydrological model evaluation across 384 catchments in Switzerland (CH) and Austria (Aus) using observed meteorology. Results are reported for A) the KGE index and B) the annual peak flow bias (APFB).**. A calibration/evaluation experiment was carried out on two independent sub-periods and the results are presented for cross-evaluation and cross-calibration (results across sub-periods were pooled). The results are shown for two snow fraction groups. Results for the evaluation period for Austria are only shown for the daily resolution because of the available time span of overlapping hourly streamflow and meteorological observations for Austria.

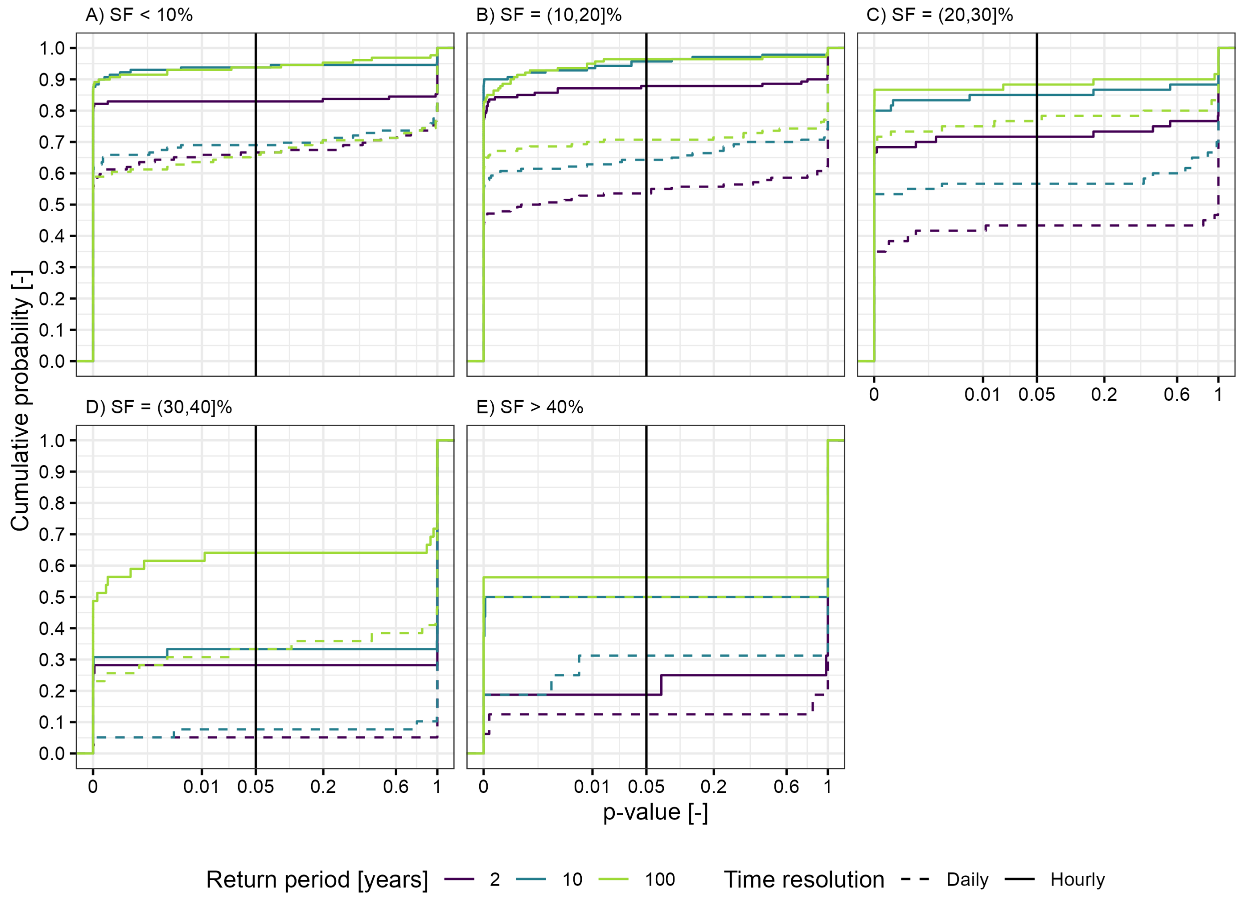

Figure S4: **Significance of the flood change signals shown in Figure 1A.** The test corresponds to a one-sided hypothesis that future flood magnitudes are **larger** than historical magnitudes. SF is snow fraction. The x-axis displays the p-values on a fourth-root Box-Cox transformed scale.

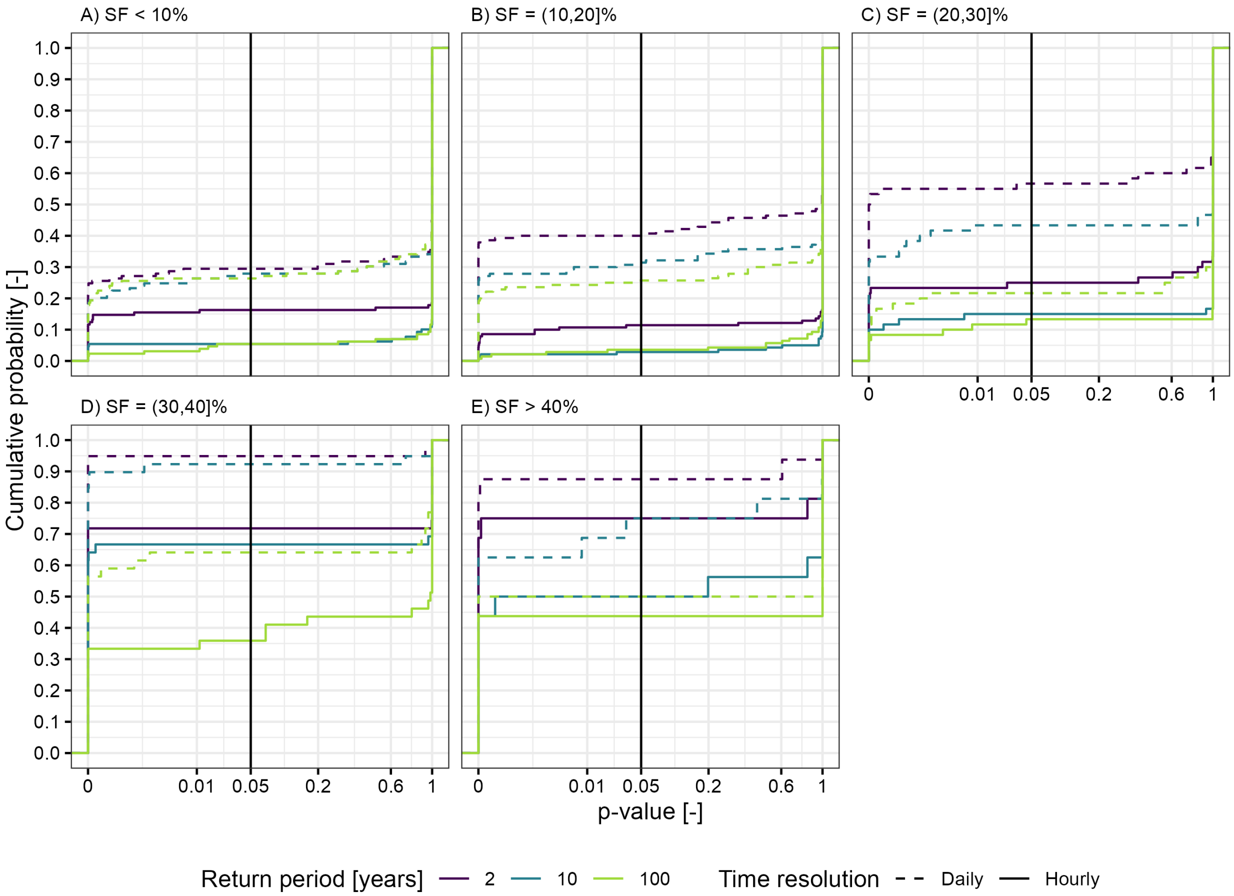

Figure S5: **Significance of the flood change signals shown in Figure 1a.** The test corresponds to a one-sided hypothesis that future flood magnitudes are **smaller** than historical magnitudes. SF is snow fraction. The x-axis displays the p-values on a fourth-root Box-Cox transformed scale.

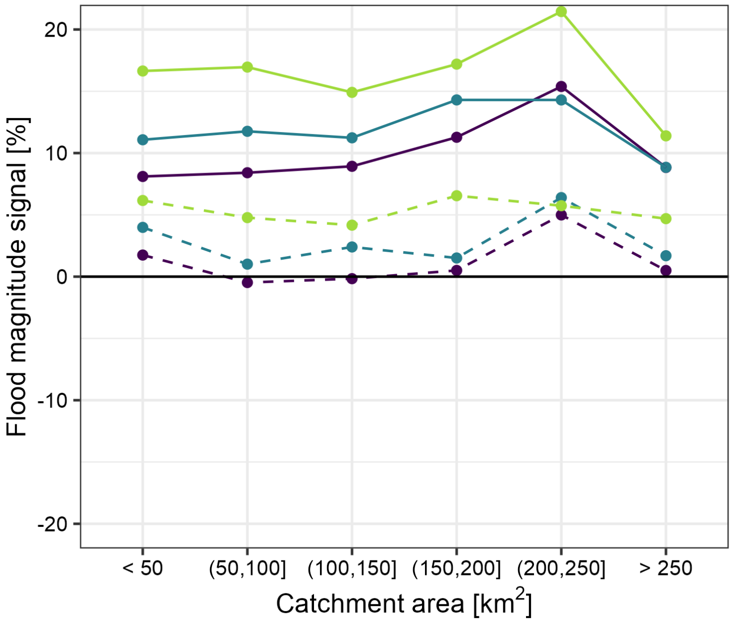

Figure S6: **Median flood magnitude change signal across catchments grouped by area.** Relates to Figure 1A in the main manuscript. The sample size for the different area classes is reported in Figure S1C.

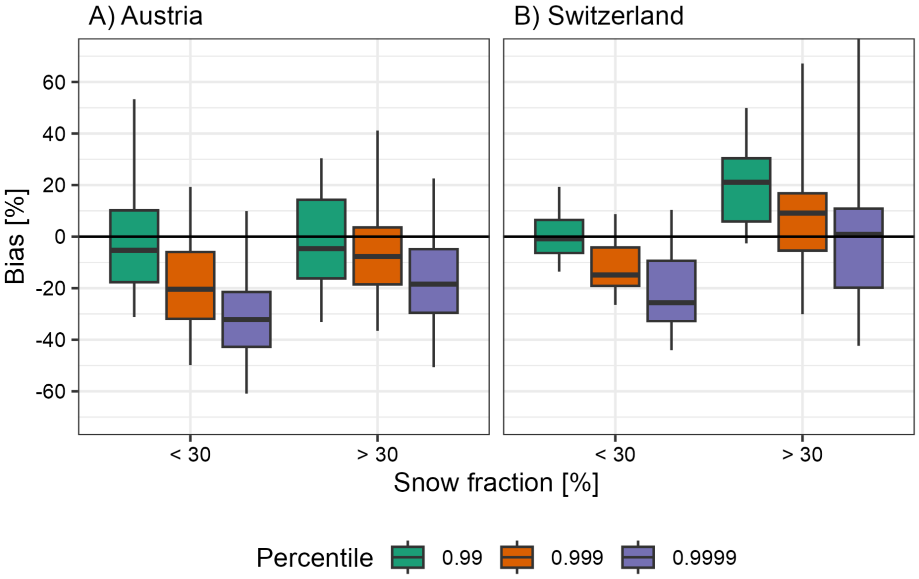

Figure S7: **Accuracy of the hourly hydrological model run with climate projections in reproducing hourly streamflow percentiles.** The percentiles of the simulations were estimated by pooling all 50 members together over the observation period.

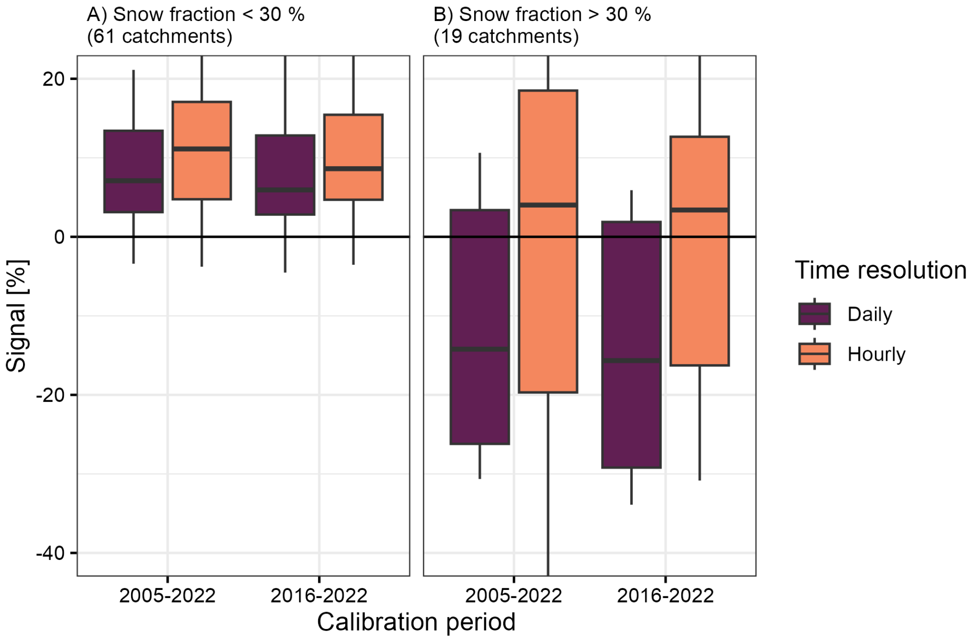

Figure S8: **Comparison between hourly and daily 100-yearly flood magnitude change signals for 80 catchments in Switzerland using two time periods of different length for the calibration of the hydrological model.**
